# Assessment of Bronchodilator Response in Patients with CF and Non-CF Bronchiectasis—A Randomized Controlled Study

**DOI:** 10.3390/jcm14134778

**Published:** 2025-07-07

**Authors:** Mordechai Pollak, Ronen Bar-Yoseph, Moneera Hanna, Noa Serruya, Guy Gut, Lea Bentur, Michal Gur

**Affiliations:** 1 Pediatric Pulmonary Institute, Rappaport Children’s Hospital, Rambam Healthcare Campus, P.O. Box 9602, Haifa 3109601, Israel; morduchp@gmail.com (M.P.); r_bar-yoseph@rambam.health.gov.il (R.B.-Y.); m_hanna@rambam.health.gov.il (M.H.); noa.serruya@campus.technion.ac.il (N.S.); g_gut@rambam.health.gov.il (G.G.); l_bentur@rambam.health.gov.il (L.B.); 2 The Adelson School of Medicine, Ariel University, Ariel 40700, Israel; 3 Faculty of Medicine, Technion Israel Institute of Technology, Haifa 32000, Israel

**Keywords:** bronchodilator response, cystic fibrosis, primary ciliary dyskinesia, bronchiectasis

## Abstract

**Objectives:** Although patients with bronchiectasis tend to have obstructive nonreversible lung functions, some have bronchodilator response (BDR), and a relatively large number are treated with bronchodilators. We assessed BDR in patients with cystic fibrosis (CF) and other bronchiectatic diseases and healthy controls (HCs) in a randomized controlled setup. **Methods:** Patients with cystic fibrosis (CF), primary ciliary dyskinesia (PCD) and non-CF non-PCD bronchiectasis (non-CF/PCD), as well as HCs, were recruited. Participants were randomly assigned to receive salbutamol (four puffs) and then a placebo or a placebo and then salbutamol. BDR was calculated using the American Thoracic Society (ATS)/European Respiratory Society (ERS) standard, defined as the change related to the individual’s predicted value (new method) or to the initial value (old method). **Results:** Sixty-nine patients (CF = 30, PCD = 20, non-CF/PCD = 19) and 20 HCs were included. Patients with CF and PCD (but not non-CF/PCD) had a statistically greater mean response to salbutamol compared with the placebo, (CF–salbutamol first: 2.82 vs. 0.85%, *p* = 0.01; placebo first: 2.39 vs. −0.27%, *p* = 0.02; PCD–salbutamol first: 5.32 vs. 1.88%, *p* = 0.01; placebo first: 2.24 vs. 0.77%, *p* = 0.05). Few patients had significant BDR (new method, >10%)—CF (0), PCD (2), non-CF/PCD (0) and HCs (2)): using the old method, an additional PCD patient and three non-CF/PCD had significant BDR (>12%). **Conclusions:** Significant BDR seems to be rare in patients with bronchiectasis. In CF and PCD, the response was greater than the placebo; the clinical significance of this difference and its therapeutic implications, as well as the best method to determine BDR, have yet to be determined.

## 1. Introduction

Bronchiectasis (BE) is a chronic respiratory condition characterized by the irreversible dilation and widening of the bronchial tubes. This structural alteration often results in persistent cough, daily sputum production and recurrent respiratory infections, significantly impairing patients’ health and quality of life [1]. BE can be the consequence of several chronic lung diseases, many of which begin in early childhood. In cystic fibrosis (CF), BE results primarily from impaired electrolyte and water transport across epithelial surfaces, which leads to thickened mucus and chronic bacterial infection [2]. Primary ciliary dyskinesia (PCD) is characterized by defective mucociliary clearance due to abnormal ciliary structure or function [3]. In immune deficiencies, BE may arise from recurrent infections or aberrant inflammatory responses [4]. Regardless of the particular cause, the clinical outcome is frequently a progressive decline in lung function, typically showing an obstructive pattern on spirometry [5].

The use of bronchodilators in the management of BE is widespread, although supporting evidence for their efficacy remains limited and somewhat controversial. Unlike asthma, in which airway hyperresponsiveness and bronchodilator response (BDR) are well-established hallmarks, the underlying mechanisms contributing to BDR in BE are less clearly understood [6,7,8,9,10,11,12,13]. Some studies suggest that airway hyperreactivity may exist in a subset of BE patients, potentially justifying bronchodilator use [14].

In addition to pharmacologic interventions, airway clearance techniques, such as those involving positive expiratory pressure (PEP), are known to facilitate mucus mobilization and improve lung function [15,16]. Spirometry, by requiring multiple forced expiratory maneuvers, may inadvertently contribute to mucus clearance, thereby enhancing post-bronchodilator spirometric values. However, the extent to which this non-pharmacological mechanism affects BDR measurements in BE has not been thoroughly investigated. In this study, our aim was to assess the degree of BDR in patients with BE and to evaluate the possible contribution of repeated forced expiration measures as a mechanism for BDR.

## 2. Methods

### 2.1. Study Design

This was a single-center, double-blind, randomized controlled study carried out in our Pediatric Pulmonary Institute between May 2023 and January 2024. Patients over five years of age, attending the clinic for regular and stable outpatient visits, were recruited. Inclusion criteria were as follows: (a) a diagnosis of CF, PCD or at least one chest computed tomography (CT) consistent with BE; (b) clinical stability, defined as the absence of a pulmonary exacerbation that required the use of systemic antibiotics up to one week prior to the study day; and (c) no use of long-acting beta agonists (LABA) 12 h before BDR testing, or short-acting beta agonist (SABA) 4 h before testing. Exclusion criteria: (a) patients under five years of age and (b) inability to perform proper spirometry.

The healthy controls (HC) were accompanying friends or family members of patients attending the outpatient clinics who agreed to participate in this study. Volunteers reporting respiratory symptoms, asthma diagnosis or treatment were excluded.

### 2.2. Randomization and Blinding

**Figure 1:**
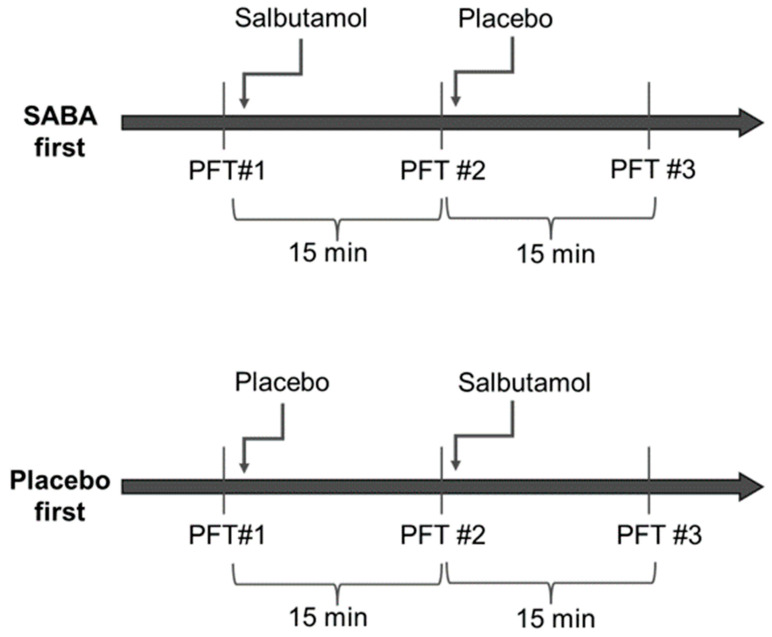
**Study design** Participants were randomized into two groups; half received salbutamol first and half received the placebo first. All participants repeated three sets of spirometry testing—at baseline, after the first round of inhalation, and after the second round of inhalation. Each set was timed to be 15 min after inhalation.

Randomization was performed for each group separately and was generated by a computer on a 1:1 basis. Half of each group was assigned to receive salbutamol first (four puffs of 100 mcg) and then the placebo (four puffs), and the other half received the placebo and then salbutamol (Figure 1). Spirometry was repeated 15 min after inhalations. Altogether, each participant had three sets of spirometry testing spaced 15 min apart. The salbutamol and placebo puffers were marked with a code and were otherwise identical and could not be distinguished by patients or staff. The clinicians, technicians and patients were not able to distinguish between salbutamol and the placebo and were blinded to the arm the patient was assigned at the time of the testing and the interpretation of the results. The department nurse was the only person in the department with access to the coded list, and she handed the inhaler to the technician performing the spirometry. All puffs were administrated via a spacer.

### 2.3. Data Collection and Pulmonary Function Tests

Demographics, anthropometrics and medication use were documented after enrolment at the time of the visit. Additional information, such as previous lab results, were obtained from the medical charts.

Spirometry was performed according to the current American Thoracic Society (ATS)/European Respiratory Society (ERS) recommendations [17]. The percentage of predicted values were calculated using the global lung initiative (GLI) equations [18]. Bronchodilation responsiveness, in terms of the change in forced expiratory volume in one second (FEV_1_), was assessed according to the 2021 ATS/ERS technical standard (new method), defined as the change related to the individual’s predicted value ($\frac{{FEV}_{1}\;\left(post\right)-{FEV}_{1}\;\left(pre\right)}{{FEV}_{1}\;\left(predicted\right)}$), and with the previous method (old method), defined as the change related to the initial value ($\frac{{FEV}_{1}\;\left(post\right)-{FEV}_{1}\;\left(pre\right)}{{FEV}_{1}\;\left(pre\right)}$) [17]. An increase of 10% after bronchodilators was considered a significant BDR when using the new method, and 12% was considered significant when using the old one.

### 2.4. Data Analyses

Descriptive statistics, including mean, standard deviation, median, percentiles and ranges, were calculated for all study parameters. The normal distribution of continuous variables was assessed using the Kolmogorov–Smirnov test. Based on these results, *t*-tests, ANOVA, Mann–Whitney, and Kruskal–Wallis tests were applied as appropriate for comparisons between groups. The effect of salbutamol was evaluated by comparing BDR results following placebo and salbutamol administration using the Wilcoxon signed-rank test within each study group. A *p*-value of <0.05 was considered statistically significant. All analyses were conducted using SPSS version 28.

The primary outcome was the response of FEV_1_ to salbutamol. An a priori power analysis was conducted with the G*Power software version 3.1.9.7 (Faul et al., 2007) to determine the minimal sample size. To detect a mean difference of 5% FEV_1_, with a power of 80% and 5% statistical significance level, a total sample size of at least 84 subjects was required. The number of healthy controls required for BDR comparisons was estimated to be at least 12 for a one-way, four-group analysis to detect a medium effect size (Cohen’s f = 0.3) at 5% significance and 80% statistical power.

### 2.5. Ethics

The institutional board reviewed and approved this study (RMB-0025-23); a written informed consent was obtained from each subject or their legal guardians prior to recruitment. This study was registered with ClinicalTrials.gov (NCT05932316).

## 3. Results

### 3.1. Baseline Characteristics

**Figure 2:**
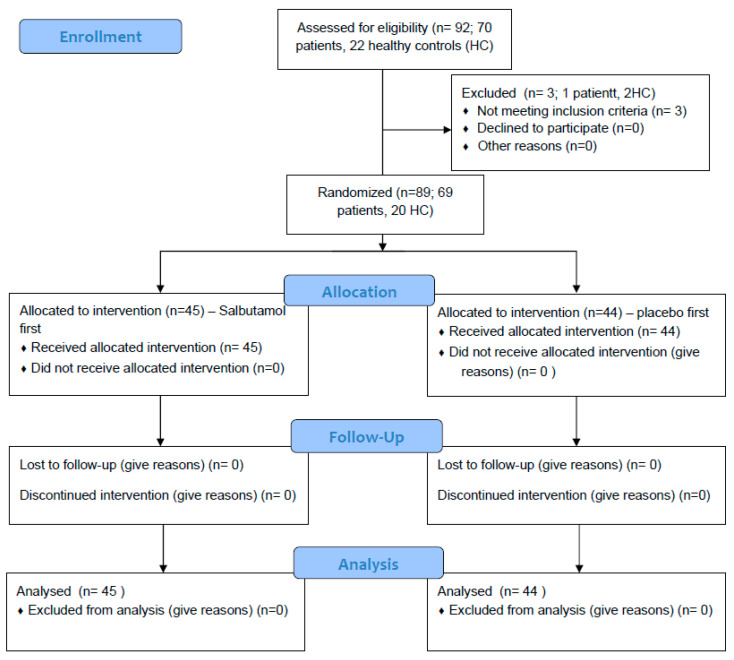
CONSORT Flow Diagram.

Out of the 92 that agreed to participate in this study, 89 were included in the analysis. One non-CF/PCD patient and two HCs were unable to perform technically acceptable spirometry on the study day and were excluded (Figure 2).

Sixty-nine patients with BE (CF = 30, PCD = 20, non-CF/PCD = 19) and 20 HCs were included in the analysis. The mean age of all participants was 18.6 ± 9.2 years; CF patients were the oldest and PCD patients were the youngest (22.4 ± 11.4 vs. 15.8 ± 6.9; *p* = 0.028). The mean FEV_1_ was not significantly different within the different BE groups (Table 1).

The CF group included 3 children (<12 years) aged 8.8 ± 2.5 years, 12 adolescents (12–17 years) aged 15.7 ± 2.0 years and 15 adults (>18 years) aged 30.4 ± 11 years; the PCD group included 6 children (8.3 ± 2.6 years), 8 adolescents (15.8 ± 1.2 years) and 6 adults (23.2 ± 6.4 years); the non-CF/PCD group included 5 children (10.2 ± 0.8 years), 6 adolescents (15.2 ± 1.7 years) and 8 adults (25.7 ± 7.7 years); and the control group included 4 children (5.12 ± 3.4 years), 10 adolescents (14.5 ± 1.6 years) and 6 adults (22.7 ± 6.9 years).

Among CF patients, 19 (63.3%) had at least one severe mutation, 15 (50%) were pancreatic insufficient and 14 (46.7%) were on modulator therapy. Of all patients, 33.3–47.4% and 38.9–68.4% were regularly using LABA and inhaled corticosteroids (ICS), respectively. Dose and duration of ICS and LABA were variable and age appropriate. Most young children received inhaled fluticasone 125 mcg via a spacer device twice daily (BID). Older children and adults received Symbicort^®^ Turbuhaler^®^ doses of 160/4.5 mcg BID. Duration was also variable and usually lasted several years; compliance was not assessed.

### 3.2. Bronchodilator Response

Table 2 presents the response to salbutamol and the placebo in the various groups. Combining all patients (n = 69), the response to salbutamol was significantly higher compared with the placebo (in both randomization groups—salbutamol/placebo first) (2.90 (1.25–6.20) vs. 0.55 (−0.63–1.51), *p* < 0.001 salbutamol first; 2.50 (1.22–3.89) vs. 0.29 (−1.12–1.72), *p* < 0.001 placebo first). For the various subgroups, a higher response to salbutamol compared with the placebo was seen in CF and PCD patients in both randomization groups but not in the non-CF/PCD group. HCs had a greater response to salbutamol, when administered before the placebo.

### 3.3. Significant BDR

**Figure 3:**
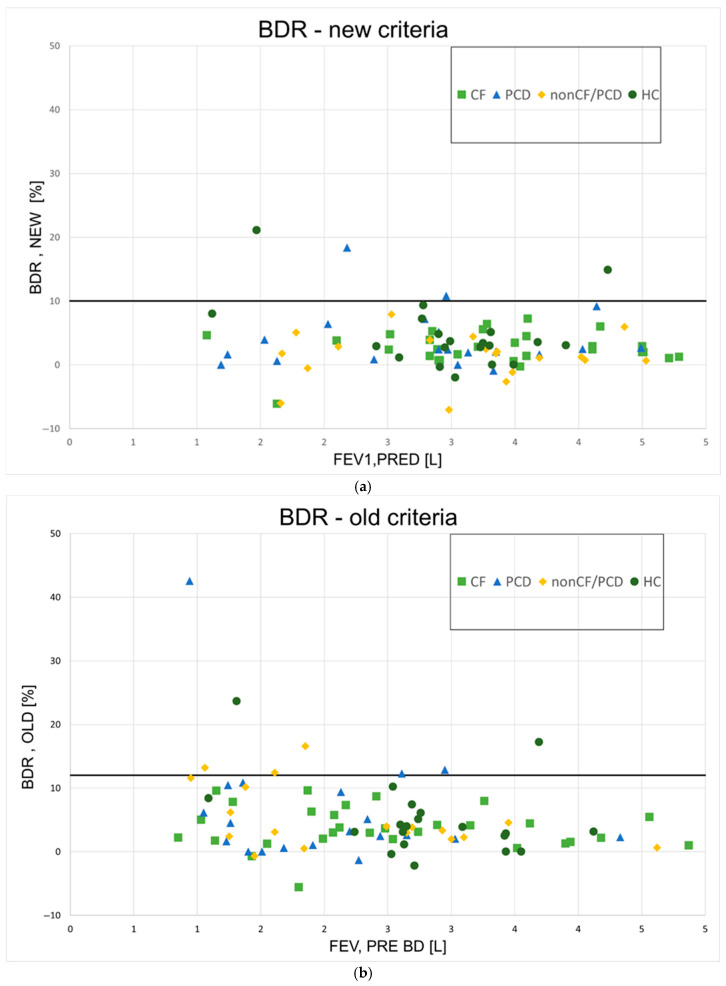
**Bronchodilator responses for all patients.** (**a**)—New ATS method; BDR = bronchodilator response; (**b**)—old ATS method; BDR = bronchodilator response; CF = cystic fibrosis; PCD = primary ciliary dyskinesia; HCs = healthy controls; L = liters.

The BDR for all participants can be seen in Figure 3 (Figure 3a—new method; Figure 3b—old method). Using the new method, four participants (3%) had a BDR over 10%, which was considered significant (two PCD, two HC); BDR was also significant for these patients when using the old method with a cutoff value of 12%. According to the old method, an additional PCD patient and three non-CF/PCD patients were found to have significant BDR. Altogether, 9% of patients had significant BDR using this method. Table 3 summarizes the number of participants with significant responses according to the new and the old methods.

## 4. Discussion

Herein, we examined BDR in people with BE in a randomized, placebo-controlled design.

Our patient population included different disease entities and pathogenetic mechanisms, all resulting in BE. Traditionally, patients with BE were classified as CF and non-CF BE. Over the last decade, the importance of differentiating PCD from other etiologies has been emphasized by the European bronchiectasis registry (EMBRAC) and the British Thoracic Society [19,20]. PCD diagnosis may affect clinical management (hearing and obstructive sleep disorder screening, fertility counseling), prognosis and genetic counselling. This separation may also enable inclusion in clinical trials, registries, future therapeutic strategies such as gene editing (CRISPER based), and ciliary-targeted treatment [21].

By comparing salbutamol to placebo, our primary objective was to assess if BDR in people with BE is attributed purely to the pharmacological effect of beta agonists. We hypothesized that additional, non-pharmacological factors might contribute to the observed changes in spirometric measures. In particular, we speculated that the process of performing repeated spirometry itself may play a role. Forced expiratory maneuvers, as required during spirometry testing, may possibly generate transient increases in intrathoracic pressure and airflow velocity, which could promote the mobilization and clearance of airway mucus. This mechanical effect may possibly mimic some of the benefits seen with physiotherapeutic techniques, such as PEP therapy, which are routinely used in BE to enhance airway clearance and improve pulmonary functions.

Although BDR was minimal and in most cases non-significant, the response to salbutamol was higher than the response to the placebo and was not different when bronchodilators were given before or after the placebo. This supports a direct effect of bronchodilators rather than the repeated forced exhalation maneuver as a cause for BDR.

The mean BDR for all patients in this study was 2.9% for the salbutamol first arm, and 2.5% for the placebo first arm. The clinical relevance of a BDR measuring less than 5% of the predicted FEV_1_ is often considered limited, as such a modest change may not translate into meaningful symptomatic improvement or altered disease trajectory. This finding further supports the concept that BE represents a primarily irreversible airway disease, characterized by fixed structural damage, regardless of the underlying etiology. The absence of substantial reversibility aligns with the notion that airway obstruction in BE is largely non-responsive to conventional bronchodilators, contrasting with diseases such as asthma in which BDR is a defining feature.

Nevertheless, it is important to consider these results in the broader context of therapeutic outcomes in chronic respiratory diseases. For example, in pivotal clinical trials that led to the approval of treatments for CF, similar magnitudes of improvement in FEV_1_ were deemed clinically significant. Dornase alfa (Pulmozyme), a mucolytic agent, was shown to increase FEV_1_ by an average of 5.8 ± 0.7% in CF patients [22]. Likewise, the use of inhaled hypertonic saline resulted in a 3.2% (range: 0.1–6.2%) improvement in FEV_1_ compared with isotonic saline [23]. We are not aware of previous studies that used a placebo-controlled design in which participants received both salbutamol and placebo during the same visit. We chose this approach to control for temporal variability in BDR due to factors like infections or seasonality. While several retrospective studies have reported on BDR in chronic lung diseases, they often assessed patients across multiple visits. For example, Levine et al. found that 39% of pwCF and 56.5% of individuals with PCD had a significant BDR at least once, but the proportion of total tests showing a response was not reported [12,24]. In contrast, Pollak et al. found that 9.4% of pediatric CF BDR tests were significant [11]. In our study, 3–9% of participants showed a significant BDR, depending on the method used, which is closer to the rate reported by Pollak. This suggests that repeated testing increases the likelihood of observing a significant response at least once. Furthermore, the relatively small sample size in our study may have reduced the statistical power to detect significant effects, increasing the risk of a type II error.

In the HC group, two participants demonstrated a significant BDR according to both evaluation methods. Although all HC participants self-reported no history of asthma or other chronic respiratory diseases, the presence of significant BDR raises the possibility that some individuals may have had undiagnosed or unreported asthma or other forms of airway hyperreactivity. Moreover, the threshold for defining a significant BDR remains a topic of ongoing debate. Current cutoff values may not fully account for the range of normal physiological variability seen in healthy individuals. Previously published data suggest that a proportion of healthy people, even without respiratory symptoms or known diseases, can exhibit BDR meeting conventional thresholds for significance [25,26]. Moreover, the coexisting of PCD and asthma was described recently, with an adjusted odd ratio of 22.3 (95% CI 10.8–45.9, *p* < 0. 001) [27].

The most recent ATS/ERS technical standard that was published in 2022 introduced a key revision to the methodology used for assessing BDR. Specifically, this guideline recommended reporting BDR as the absolute change in FEV_1_ or FVC expressed as a percentage of the predicted value (new method), rather than as a percentage of the pre-bronchodilator FEV_1_ or FVC (old method). This adjustment was based on findings from adult population studies, suggesting that the new method reduces the influence of anthropometric variables—such as sex and height—on BDR interpretation [17,26,28,29]. By standardizing measurements relative to predicted values, this approach aims to offer a more equitable assessment of true airway responsiveness across diverse patient groups. However, this methodological shift has not yet been thoroughly validated in pediatric populations, who differ significantly from adults in terms of growth, lung development and disease expression. Additionally, the application of this method in BE—a heterogeneous condition with variable baseline obstruction and response to therapy—has not been widely studied.

It should also be noted that the old method is widely used to help differentiate asthma (often reversible) from chronic obstructive pulmonary disease (COPD, less reversible). The new method emphasizes graded or continuous assessment; thus, there is a shift from a binary response (positive/negative) to a clinical interpretation, as even a small response in a BE patient may still be meaningful.

In our study, we evaluated BDR using both the new and old methods. While mean BDR values did not differ significantly at the group level, we observed that a greater number of participants were classified as having a significant BDR using the old method. This finding raises important questions about which method may be more sensitive—or which potentially overestimates—airway reversibility in BE. Recently, Hatic et al. found that the implementation of the ERS/ATS 2022 guidelines appears to underestimate the prevalence of BDR [30].

Therefore, further large-scale, longitudinal studies are warranted to clarify which calculation method best reflects clinically meaningful BDR in both pediatric and adult populations with BE.

Finally, the clinical implications of BDR in BE should be discussed. As Oscullo et al. recently found, inhaled bronchodilators are widely used in BE, despite sparse evidence from randomized controlled trials (RCT). Bronchodilators may provide modest lung function improvement in a few BE patients [31]. In view of this limited data, the use of bronchodilators should be limited to patients demonstrating BDR > 10% or clinical benefit. Children with PCD may need to be evaluated for coexisting asthma. Further studies evaluating the benefit of short-acting beta-agonists, LABA and long-acting muscarinic agonists (LAMA) are needed, especially in children.

The main strength of this study lies in its prospective, randomized controlled design for assessing BDR. Most studies on BDR in BE are retrospective and therefore subject to bias. Additionally, it evaluates BDR across different BE types and includes comparisons with HCs.

However, this study is limited by a relatively small cohort size, which, combined with the subtle differences observed between patients and controls, reduces its statistical power. The small number of participants exhibiting significant BDR precluded subgroup comparisons. The group of patients is heterogeneous in age, disease severity and mechanisms leading to BE; this heterogeneity may lead to different and variable mechanisms of BDR. The use and effectiveness of bronchodilators in BE may differ between children and adults due to differences in pathophysiology, disease duration, comorbidity and available evidence. However, the small number of children (<12 years old) limited our ability to perform sub-analysis. Larger studies are needed to better characterize and distinguish patients with significant BDR.

## 5. Conclusions

Our findings indicate that while spirometric improvements following salbutamol administration were modest and largely below the traditional threshold of clinical significance, they were nonetheless greater than those observed with the placebo. This suggests that the pharmacologic action of beta-agonists plays a more prominent role than non-specific effects such as repeated forced expiratory maneuvers. This study also raises important questions regarding the optimal method for calculating BDR in both pediatric and adult BE populations, as current standards may not fully account for disease-specific or developmental factors. Larger longitudinal studies are needed to further refine diagnostic criteria, explore response variability across BE subtypes and determine which patients may derive the greatest benefit from bronchodilator therapy.

## Figures and Tables

**Table 1:** Patient characteristics.

	All (n = 89)	CF (n = 30)	PCD (n = 20)	Non-CF/PCD (n = 19)	HCs (n = 20)
Age (years)	18.6 ± 9.2	22.4 ± 11.4	15.8 ± 6.9	18.3 ± 8.4	15.9 ± 6.4
BMI (kg/m^2^)	21.1 ± 4.7	20.8 ± 4.3	22.1 ± 5.9	19.7 ± 3.5	22 ± 4.9
Male (%)	40.4	46.7	30	36.8	40.9
FEV_1_^a^ (%)	78.1 ± 22	74.8 ± 23.8	75 ± 22.9	70.2 ± 20.9	93.7 ± 9.1
FVC ^a^ (%)	80.4 ± 17.7	79.9 ± 18.1	78.9 ± 20.2	72.9 ± 18.7	89.8 ± 7.7
FEF_25–75_^a^ (%)	75.2 ± 34.2	69 ± 37.4	69.2 ± 32.7	64.4 ± 28.3	100.7 ± 23.4
LABA ^b^ (%)	39.8	40	45	33.3	-
ICS ^b^ (%)	48.5	43.3	65	38.9	-
IgE (kU/L)	(n = 52) 32.7	(n = 26) 26.4	(n = 15) 36	(n = 11) 50.1	-
	[16.6–94.6]	[20.9–143.8]	[15.3–90.8]	[10.4–255]	-
Eos (cells/µL)	(n = 58) 150	(n = 26) 150	(n = 19) 100	(n = 13) 260	-
	[87.5–252.5]	[87.5–230]	[60–210]	[115–350]	-

Values are presented as mean ± SD for all values except for IgE and Eosinophils, which are presented in median IQR. CF, cystic fibrosis; PCD, primary ciliary dyskinesia; HCs = healthy controls; BMI, body mass index; FEV_1_, forced expiratory volume in one second; FVC, forced vital capacity; FEF_25–75_, forced expiratory flow between 25% and 75% of vital capacity; LABA, long-acting beta-agonists; ICS, inhaled corticosteroids; IgE, immunoglobulin E; Eos, eosinophils. ^a^ Spirometry values at baselines (first spirometry) are presented as percents of expected, according to GLI reference values. ^b^ Portion of patients who reported daily use of inhalers.

**Table 2:** Response to bronchodilators and placebo.

	Randomization	BDR ^a^ (%)	Response to Placebo ^b^ (%)	*p* Value
CF (n = 30)	Salbutamol first (n = 15)	2.86 (1.25–5.54)	0.56 (−0.28–1.56)	0.015
	Placebo first (n = 15)	2.39 (1.06–3.43)	0.00 (−0.15–1.03)	0.008
PCD (n = 20)	Salbutamol first (n = 9)	2.67 (0.61–9.18)	0.67 (−0.61–2.10)	0.016
	Placebo first (n = 11)	2.09 (1.22–3.20)	0.74 (−0.98–2.21)	0.05
Non-CF/PCD (n = 19)	Salbutamol first (n = 10)	2.08 (0.85–4.15)	1.19 (0.00–1.96)	0.17
	Placebo first (n = 9)	2.81 (1.34–5.55)	1.91 (−1.52–3.40)	0.33
All patients ^c^ (n = 69)	Salbutamol first	2.90 (1.25–6.20)	0.55 (−0.63–1.51)	<0.001
	Placebo first	2.50 (1.22–3.89)	0.29 (−1.12–1.72)	<0.001
HCs (n = 20)	Salbutamol first (n = 10)	3.46 (2.18–7.75)	−1.03 (−2.25–0.65)	0.017
	Placebo first (n = 10)	2.91 (0.87–5.86)	0.10 (−1.4–2.65)	0.059

Values are presented as median (IQR); BDR = bronchodilator response. The effect of salbutamol was evaluated by comparing BDR results following the placebo and salbutamol administration using the Wilcoxon signed-rank test within each study group. CF = cystic fibrosis; PCD = primary ciliary dyskinesia; HCs = healthy controls. ^a^ BDR was calculated according to the ATS 2021 method $=\frac{{FEV}_{1}\;after\;BD-{FEV}_{1}\;before\;BD}{\;{FEV}_{1}\;predicted}*100$; ^b^ The response to the placebo was calculated using the same method as BDR. ^c^ Patients = CF + PCD + non-CF/PCD.

**Table 3:** Significant bronchodilator responses.

	All Patients	CF	PCD	Non-CF/PCD	HCs
n	69	30	20	19	20
New ATS/ERS method	2 (3%)	0 (0%)	2 (10%)	0 (0%)	2 (10%)
Old ATS/ERS method	6 (9%)	0 (0%)	3 (15%)	3 (16%)	2 (10%)

Bronchodilator response was calculated with both the 2021 method (new ATS/ERS method) and the previous method (old method). When using the new method, a 10% response was considered significant; for the old method, 12% was considered significant. CF, cystic fibrosis; PCD, primary ciliary dyskinesia; HCs = healthy controls; ATS = American Thoracic Society.

## Data Availability

The data presented in this study are available upon request from the corresponding author.
